# Frequency of conjugative transfer of plasmid-encoded *ISEcp1 *- *bla*_CTX-M-15 _and *aac(6')-lb-cr *genes in *Enterobacteriaceae *at a tertiary care center in Lebanon - role of transferases

**DOI:** 10.1186/1476-0711-9-19

**Published:** 2010-07-20

**Authors:** Mohamad Harajly, Marie-Therese Khairallah, John E Corkill, George F Araj, Ghassan M Matar

**Affiliations:** 1 Department of Microbiology and Immunology, Faculty of Medicine, American University of Beirut, Beirut, Lebanon; 2 Department of Pathology and Laboratory Medicine, Faculty of Medicine, American University of Beirut, Beirut, Lebanon; 3 Department of Medical Microbiology University of Liverpool, Liverpool, UK

## Abstract

**Background:**

The frequency of transfer of genes encoding resistance to antimicrobial agents was determined by conjugation in ESBL-producing and/or fluoroquinolone or aminoglycoside resistant Enterobacteriaceae clinical isolates at a tertiary care center in Lebanon. In addition, the role of *tra *genes encoding transferases in mediating conjugation was assessed.

**Methods:**

Conjugation experiments were done on 53 ESBL-producing and/or fluoroquinolone resistant *E. coli *and *K. pneumoniae *and ESBL-producing *S. sonnei *isolates. Antimicrobial susceptibility testing on parent and transconjugant isolates, and PCR amplifications on plasmid extracts of the resistance-encoding genes: *bla*_CTX-M-15 _with the *ISEcp1 *insertion sequence, the *aac(6')-lb-cr *and *qnr*S genes, as well as *tra *encoding transferases genes were done. Random amplified polymorphic DNA (RAPD) analysis was performed to demonstrate whether conjugative isolates are clonal and whether they are linked epidemiologically to a particular source.

**Results:**

Antimicrobial susceptibility testing on transconjugants revealed that 26 out of 53 (49%) ESBL-producing *Enterobacteriaceae *were able to transfer antimicrobial resistance to the recipients. Transfer of high-level resistance to the transconjugants encoded by the *bla*_CTX-M-15 _gene downstream the *ISEcp1 *insertion sequence against 3rd generation cephalosporins, and of low-level resistance against ciprofloxacin, and variable levels of resistance against aminoglycosides encoded by *aac(6')-lb-cr *gene, were observed in transconjugants. *tra *encoding transferase genes were detected exclusively in conjugative isolates.

**Conclusion:**

In conclusion, the frequency of transfer of antimicrobial resistance in non clonal *Enterobacteriaceae *at the tertiary care center by conjugation was 49%. Conjugation occurred in isolates expressing the *tra *encoding transferase genes. Multiple conjugative strains harboring the plasmid encoded antimicrobial resistant genes were circulating in the medical center. Molecular epidemiology analysis showed that conjugative isolates are neither clonal nor linked to a particular site and transfer of antimicrobial resistance is by horizontal transfer of plasmids.

## Background

Plasmid-encoded Extended-spectrum β-lactamases (ESBL) are increasingly spreading among *Enterobacteriaceae *clinical isolates throughout the world due mostly to their presence on highly conjugative plasmid. Surveys that were done in Canada, Greece, United Kingdom and Italy showed an association between the CTX-M type ESBL and resistance to other antimicrobial agents [1]. This was explained by a number of findings showing that *bla*_*CTX-M *_genes are commonly found on large plasmids that often carry other genes conferring resistance to other antimicrobial agents including aminoglycosides, fluoroquinolones, chloramphenicols, tetracyclins and others (particularly, *bla*_*OXA-1*_*, bla*_*TEM-1*_*, tetA, aac(6')-lb-cr) *[2,3]. CTX-M-15, one of the CTX-M enzymes which has an increased catalytic activity against ceftazidime[3], is now found worldwide especially in *E. coli *isolates from France, Canada and the United Kingdom [3-6].

pC15-1a, one of the plasmids responsible for the spread of the *bla*_*CTX-M-15 *_gene, was found associated with the Canadian outbreak, and its complete sequence was reported by Boyd et.al [4]. This plasmid was also found to harbor in addition to the *bla*_*CTX-M-15 *_gene, the aminoglycoside modifying enzyme encoding gene, *aac(6')-lb-cr*, which confers resistance to two unrelated antimicrobial agent classes, the aminoglycosides and the fluoroquinolones, by acetylating these drugs. The mechanism of resistance to fluoroquinolones encoded by *aac(6')-lb-cr *gene is different from that induced by other fluoroquinolones resistance encoding genes such as *qnr *genes [7]. In addition the role of plasmid mediated *tra *genes encoding transferase proteins was assessed.

In Lebanon, 96% of ESBL-producing *E.coli *and *K.pneumoniae *clinical isolates from various sources harbored *bla*_*CTX-M-15 *_and a number of these isolates carried also *aac(6')-lb-cr *gene [8]. The encoding genes of the two enzymes were found on pC15-1a, and other related plasmids. The *bla*_*CTX-M-15 *_gene located on these plasmids was found downstream of the *ISEcp1 *insertion sequence implicated in its expression [9]. A recent study[10] done on *Shigella sonnei *isolates revealed that 4 ESBL-producers had a plasmid harboring *bla*_*CTX-M-15 *_gene with the *ISEcp1 *but had no resistance to floroquinolones.

Based on the results of these studies and others, we aimed at determining the frequency of conjugation in 53 ESBL-producing and/or fluoroquinolone resistant *E.coli *and *K.pneumoniae *and 4 ESBL-producing *S.sonnei *isolates from a major tertiary care center, and confirming transfer of resistance to recipients by antimicrobial susceptibility testing and PCR amplification of the encoding resistance genes, *ISEcp1*-*bla*_*CTX-M-15*_, *aac(6')-lb-cr *and *qnrS*. In addition the role of plasmid encoded *tra *genes encoding transferase proteins in the transfer of antimicrobial resistance was investigated.

## Methods

### Bacterial strains

Fifty-three clinical isolates of ESBL producing *E. coli *(n = 25), *K. pneumoniae *(n = 24) and *S. sonnei *(n = 4) were collected at the Medical center and identified to the species level using the API 20E (Biomerieux, Marcy L'Etoile, France). These isolates were used as donor or parent strains in conjugation experiments.

### Conjugation experiments

Parental strains and J53 *E.coli *were conjugated in Luria Bertani broth (Becton, Dickinson and company-BBL^®^) using 1:10 ratio. The transconjugants were selected using Mueller-Hinton II (Becton, Dickinson and company-BBL^®^) agar plates containing 100 mg/l sodium azide plus 2 mg/l cefotaxime[8].

### Antimicrobial susceptibility testing

MICs of the antibiotics ceftazidime, cefpodoxime, cefotaxime, ciprofloxacin, gentamicin and kanamicin, for the parental strains and the transconjugants were determined using the E-test strips (AB Biodisk, Solna, Sweden). ESBLs detection was done by the combination disk method according to the CLSI guidelines [5], using disks containing ceftriaxone or ceftazidime with clavulanic acid (30/10 μg) and disks containing ceftazidime (30 μg) or ceftriaxone (30 μg) alone. ESBLs production was confirmed by a zone of inhibition for the combination disk that is at least 5 mm larger than that of the cephalosporin alone.

### Plasmid extraction

Qiagen plasmid mini kit (GmbH, Hilden, Germany) was used to extract plasmids from the parental strains and their transconjugants according to manufacturers' specifications.

### Polymerase Chain Reaction (PCR)

Primers were selected to amplify the *aac(6')-lb-cr *[11] and *qnrS *[12] genes, and the sequence containing the ISEcp1 insertion sequence and part of the *bla*_*CTX-M-15 *_gene (CTX-MA3 (5'-ACY TTA CTG GTR CTG CAC AT-3'), and the forward primer of the mobilizing insertion sequence ISEcp1U1 (5'-AAA AAT GAT TGA AAG GTG GT-3) as described previously [13] as well as the whole *bla *_CTX-M-15 _gene using the following primers: CTX-M-15-F (5'-GGTTAAAAAATCACTGCGTC-3') and CTX-M-15-R (5'-TTACAAACCGTCGGTGACGA-3'). The *bla *_CTX-M-15 _gene primers aligned with positions 3-876 of accession number FJ815288. Primers of all genes were used for PCR amplification on the extracted plasmids from the parents and the transconjugants. In addition primers amplifying the *tra *genes encoding the transferase proteins TraM, TraY, TraI to OriT, [14,15] and TraD [16] was performed on conjugative and non-conjugative isolates. PCR products were run on 1.5% agarose gel, stained with ethidium visualized under U.V. light and photographed using the dig-it-doc it software and Olympus digital camera. Sequence analysis was performed on all amplicons in a previous study [8].

### Random amplified polymorphic DNA (RAPD)

RAPD analysis [8] was performed using **t**he Ready-To-Go kit (GE healthcare, Buckinghamshire, UK ) according to manufacturer's specifications. Gels were analyzed by the BIONUMERICS (Applied Maths, Sint-Martens-Latem, Belgium).

## Results

**Figure 1:**
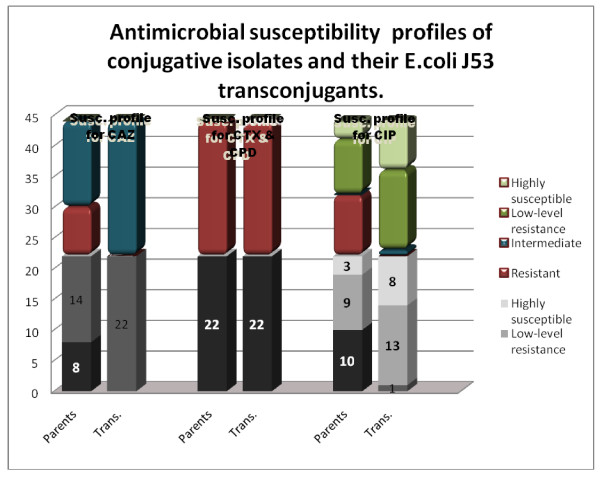
**Antimicrobial susceptibility profiles done on the 22 conjugative isolates (parents) and their transconjugants for the antimicrobial agents, ceftazidime (CAZ), cefotaxime (CTX), cefpodoxime (CPD) and ciprofloxacin (CIP)**.

Our data showed that 26 of 53 (49%) *E. coli *and *K. pneumonia*e isolates were able to transfer resistance to recipients (Table 1). E-test done on 22 of the conjugative isolates showed that all of them expressed high-level resistance (with MIC of more than 256 μg/ml) to cefotaxime and cefpodoxime and were able to transfer this high-level resistance to the recipient strain. Eight and 14 of 22 revealed high-level resistance and intermediate resistance (MIC values between 16 and 32 μg/ml) to ceftazidime respectively; twenty one of the transconjugants expressed intermediate resistance and 1 revealed low-level resistance to ceftazidime (MIC equals to 8 μg/ml). Concerning resistance profile to ciprofloxacin, 10 and 9 out of the 22 isolates revealed high-level resistance (MIC of more than 32 μg/ml) and low-level resistance (MIC values ranging between 0.064 and 0.25 μg/ml, slightly higher than the MIC for the J53 *E.coli *before conjugation which is 0.047 μg/ml) respectively. Eight of the 10 resistant and 5 out of the 9 isolates with low-level resistance transferred low-level resistance against ciprofloxacin to the recipient strain. Only one transconjugant revealed intermediate resistance to ciprofloxacin with an MIC of 3 μg/ml (Figure 1, Table 1, 2).

**Table 1:** MIC (μg/ml) of the antimicrobial agents, ceftazidime (CAZ), cefotaxime (CTX), cefpodoxime (CPD), ciprofloxacin (CIP), gentamicin (GEN) and kanamicin (KAN), and PCR amplification results of the resistance-encoding genes on selected conjugative isolates and their corresponding *E. coli *J53 (sodium azide-R) transconjugants, in comparison to their RAPD profiles.

**Isolate number (parents and transconjugants)**	**MIC of antimicrobial agents**						**Resistance-encoding genes**			**RAPD profile**
	**CAZ**	**CPD**	**CTX**	**CIP**	**GEN**	**KAN**	***aac (6')lb-cr***	**ISEcp1-*bla*_*CTX-M-15*_**	***qnrS.***	
***E. coli *J53**	**0.19**	**0.064**	**0.75**	**0.047**	**0.5**	**1.5**	-	-	-	
***E. coli *1**	>256	>256	>256	>32	96	12	+	+	+	E-d
***E. coli *1 T**	32	>256	>256	3	16	6	+	+	+	
***E. coli *3**	16	>256	>256	0.125	32	12	+	+	-	E-j
***E. coli *3 T**	16	>256	>256	0.125	16	8	+	+	-	
***E. coli *6**	24	>256	>256	>32	1.5	16	+	+	-	E-l
***E. coli *6 T**	16	>256	>256	0.125	0.75	6	+	+	-	
***E. coli *8**	16	>256	>256	>32	128	32	+	+	-	E-r
***E. coli *8 T**	16	>256	>256	0.094	12	8	+	+	-	
***E. coli *11**	24	>256	>256	>32	>256	64	+	+	-	E-l
***E. coli *11 T**	16	>256	>256	0.125	16	16	+	+	-	
***E. coli *12**	24	>256	>256	>32	64	24	+	+	-	E-l
***E. coli *12 T**	12	>256	>256	0.125	16	4	+	+	-	
***E. coli *14**	16	>256	>256	0.094	16	3	+	+	-	E-b
***E. coli *14 T**	8	>256	>256	0.047	1.5	6	+	+	-	
***E. coli *15**	24	>256	>256	>32	1.5	24	+	+	-	E-q
***E. coli *15 T**	24	>256	>256	0.125	1.5	4	+	+	-	
***K. pneumoniae *1**	>256	>256	>256	>32	128	48	+	+	-	K-e
***K. pneumoniae *1 T**	32	>256	>256	0.125	16	8	+	+	-	
***K. pneumoniae *2**	32	>256	>256	0.25	>256	32	+	+	-	K-o
***K. pneumoniae *2 T**	16	>256	>256	0.125	12	8	+	+	-	
***K. pneumoniae *13**	96	>256	>256	0.5	1.5	4	+	+	-	K-g
***K. pneumoniae *13 T**	24	>256	>256	0.047	0.5	4	+	+	-	
***S. sonnei *1**	12	>256	>256	0.047	2	8	-	+	-	S-a
***S. sonnei *1 T**	12	>256	>256	0.047	0.5	1.5	-	+	-	
***S. sonnei *3**	12	>256	>256	0.047	2	8	-	+	-	S-a
***S. sonnei *3 T**	12	>256	>256	0.047	0.5	1.5	-	+	-	
***S. sonnei *4**	12	>256	>256	0.047	2	8	-	+	-	S-a
***S. sonnei *4 T**	12	>256	96	0.047	0.5	1.5	-	+	-	

**Table 2:** RAPD profiles of non-conjugative isolates.

**Isolate number**	**RAPD profile**
***E. coli *2**	E-e
***E. coli *4**	E-r
***E. coli *5**	E-f
***E. coli *7**	E-r
***E. coli *9**	E-s
***E. coli *10**	E-k
***E. coli *13**	E-q
***E. coli *16**	E-l
***E. coli *17**	E-k
***E. coli *18**	E-m
***E. coli *19**	E-c
***E. coli *23**	E-j
***E. coli *21**	E-d
***E. coli *22**	E-c
***E. coli *23**	E-j
***K. pneumoniae *3**	K-o
***K. pneumoniae *4**	K-m
***K. pneumoniae *7**	K-g
***K. pneumoniae *8**	K-g
***K. pneumoniae *12**	K-n
***K. pneumoniae *14**	K-g
***K. pneumoniae *16**	K-g
***K. pneumoniae *21**	K-n
***K. pneumoniae *22**	K-h
***K. pneumoniae *24**	*K-i*

PCR detection confirmed the transfer of the *bla*_*CTX-M-15 *_gene together with the *ISEcp1 *insertion sequence and the *aac(6')-lb-cr *gene from the parental strains to their recipients. Only one isolate harbored the *qnrS *gene and was able to transfer it together with the *aac(6')-lb-cr *gene to its recipient that expressed intermediate resistance to ciprofloxacin (Table 1). PCR amplification of the *tra *genes was positive exclusively in the conjugative isolates.

**Figure 2:**
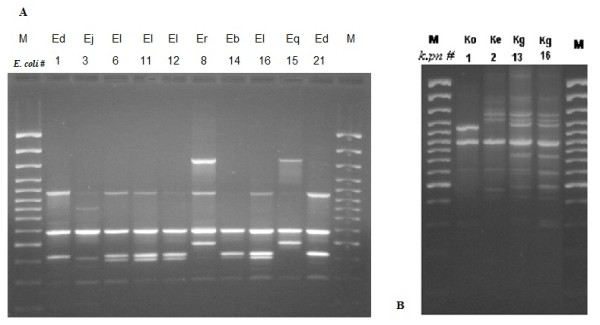
**A Representative RAPD gel on *E. coli *isolates (RAPD types Ed, Ej, El Er Eb El Eq, Ed with corresponding numbers in Table 1&2)**. M: 100 bp ladder. B Representative RAPD gel on *K. pneumoniae *isolates (RAPD types Ko, Ke, Kg, with corresponding numbers in Tables 1&2)

**Figure 3:**
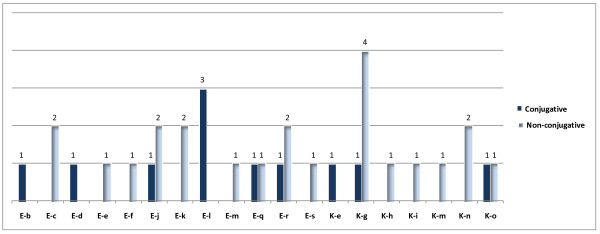
**Histogram showing the frequency of conjugative and non-conjugative isolates corresponding to each RAPD profile**.

RAPD analysis (Table 1, Figures 2A,B, 3) showed that *E. coli *and *K. pneumoniae *conjugative isolates are not clonal. Multiple strains are common to both conjugative and non-conjugative isolates. *S. sonnei *isolates are clonal. Epidemiological data in conjunction with molecular analysis confirmed that transfer of resistance among conjugative strains is merely by horizontal transfer of antimicrobial resistance plasmid encoded determinants and that strains were linked to multiple sources.

## Discussion

In this study, we determined the frequency and mode of transfer of resistance to antimicrobial agents by conjugation, among a number of multi-drug resistant *Enterobacteriaceae *isolates. These are all ESBL producing and/or fluoroquinolone and aminoglycoside resistant. Most of these isolates harbor the pC15-1a plasmid, that carries the *bla*_*CTX-M-15 *_gene in addition to the aminoglycoside modifying enzyme *aac(6')lb-cr *gene which also confers some resistance to the fluoroquinolone ciprofloxacin as demonstrated in a previous study [8]. The MDR region is [8] indeed harbored on the pC151a or the pCTX15 plasmids since first, the plasmids characterization done in the previous study showed that the hpa1 digestion profile was related to the pC151a or the pCTX15 plasmids and second the PCR based detection of the *bla *_CTX-M-15 _and the aac6'lb-cr genes on transferable plasmids in transconjugants were on pC15-1a or the pCTX-15 plasmids.

Molecular epidemiology analysis confirmed that conjugative isolates are not clonal and transfer of antimicrobial resistance was acquired through horizontal transfer of plasmids. Besides conjugative strains were linked epidemiologically to multiple sources. No single conjugative strain harboring the plasmid encoded antimicrobial resistant genes was circulating in the medical center.

Conjugation experiments in this study have shown that about 50 percent of the *Enterobacteriaceae *isolates were able to transfer resistance to cefotaxime, which is a significantly high prevalence rate of transfer of resistance among ESBL producing multi-drug resistant isolates. As demonstrated, conjugative isolates harbored the plasmid encoded *tra *genes encoding the transferase proteins TraM responsible for mating aggregation, TraY directing the nicking enzyme TraI to OriT, [14] TraI inducing nicking and unwinding [15] and TraD involved in pumping the DNA into the recipient cells [16] and hence were able to transfer resistance.

All the isolates were resistant to the 3^rd ^generation cephalosporins, and conjugative ones were able to transfer resistance against these antimicrobial agents to the recipients. Most of the isolates were able to transfer high-level resistance against cefotaxime and cefepodoxime to the transconjugants which revealed MIC equal or above 256. It was found however, that the MIC of ceftazidime for most of the donor isolates was less than that usually reported for CTX-M-15 producing isolates (between 128 and 256 μg/ml) (1). CTX-M-15 differs from its relative CTX-M-3 by a single amino-acid substitution, which increases its activity against ceftazidime[17]. Most of the *bla*¬_*CTX-M-15 *_genes encountered in ESBL- producing isolates are located on plasmids downstream an *ISEcp1 *insertion sequence which harbors the -10 and -35 promoter sequences (TTGACA and TAAACT respectively) essential for the high expression of the gene and hence, the increased activity against ceftazidime and other third generation cephalosporins. These were detected in a previous study [8]. However, CTX-M-15-producing strains with low activity against ceftazidime were previously reported in many countries including UK and the United Arab Emirates [5,8]. A study that was done by Wooford et. al on the UK isolates, showed that there is a number of CTX-M-15-producing *E. coli *for which ceftazidime had MIC values equal or less than 32 μg/ml, which is lower than the usually reported for the CTX-M-15 producing *Enterobacteriaceae *(between 128 and 256 μg/ml) [18]. Genotypic characterization by Woodford et al.[5] demonstrated that the *bla*_*CTX-M-15 *_gene was located on a plasmid downstream the *ISEcp1*; however, an IS26 insertion element was present within the terminal inverted repeat of *ISEcp1*, separating *bla*_*CTX-M-15 *_from its usual promoter located in the *ISEcp1*. This genotypic characteristic was thought to be responsible for the lower *bla*_*CTX-M-15 *_expression, and hence, the decreased ceftazidime MIC for the UK and the UAE strains. We suspect that the same genotypic feature is also responsible for the level of resistance against ceftazidime that is lower than expected in our CTX-M-15 producing strains.

All the transconjugants on the other hand, had intermediate resistance to ceftazidime. The seven isolates that were resistant to ceftazidime with MIC above 96 were unable to transfer this resistance to the recipients, for which the MIC of ceftazidime did not cross 32 μg/ml. This could have resulted from the presence of chromosomally-mediated β-lactamase genes in the parental strains that could not be transferred to the recipients.

With respect to the transfer of resistance to ciprofloxacin, it was noticed that all except one of the transconjugants revealed low-level resistance, resulting from the transfer of *aac(6')-lb-cr*. This gene has an aminoglycoside-modifying activity, and crossed the antimicrobial agents' class boundaries by its activity also against the fluoroquinolones. Although the degree of resistance conferred is small, this gene was shown to act additively with other genes that confer resistance against fluoroquinolones, like the plasmid-mediated *qnr *genes, or chromosomal mutations. Despite the relatively low activity of the *aac(6')-lb-cr *gene product against fluoroquinolones, it could play an important role in facilitating the selection of strains with mutations in the chromosomal *gyrA, gyrB*, and *parC *genes, among a bacterial population that is exposed to be fluoroquinolone resistant[19].

The *qnr *genes are other important plasmid-mediated genes that confer resistance to fluoroquinolones. *qnrA *is the first discovered and is found worldwide. *qnrB *and *qnrS *are recently emerging, and are still not as widespread [20]. Only one of our isolates, *E. coli *1, was able to transfer intermediate-level resistance against ciprofloxacin to transconjugants, with an MIC in the transconjugant of 3 μg/ml; whereas in all the other transconjugants, ciprofloxacin had a maximum MIC value of 0.125 μg/ml. We suspected the presence of one of the plasmid-mediated *qnr *genes in *E. coli *1 in addition to the *aac(6')-lb-cr*, and indeed, PCR amplification on the plasmid extract from this isolate confirmed the presence of both genes in the donor and the transconjugant. The higher MIC of ciprofloxacin in the *E. coli *1 transconjugant compared to that of the transconjugants of the other isolates, leading to intermediate-level ciprofloxacin resistance is most probably due to the simultaneous transfer of both the *qnrS *and *aac(6')-lb-cr *genes to them, and hence, the coordinating action of 2 different fluoroquinolone-resistance mechanisms. It is known that the *qnrS *gene by itself does not confer intermediate-level resistance to ciprofloxacin [21,22].

In conclusion 49% of the *E. coli *and *K. pneumoniae *of non-clonal conjugative isolates in our medical center were able to transfer resistance to third generation cephalosporins in transconjugants as encoded by the *bla*_*CTX-M-15 *_gene along with its promoter in the *ISEcp1 *insertion element. In addition the isolates were also able to transfer a low-level resistance to ciprofloxacin and variable resistance to aminoglycosides, encoded by *aac(6')-lb-cr *gene. Conjugation occurred in isolates expressing the *tra *encoding transferase genes. Multiple conjugative strains harboring the plasmid encoded antimicrobial resistant genes were circulating in the medical center.

## Competing interests

The authors declare that they have no competing interests.

## Authors' contributions

MH participated in designing the experiments, executing them, performing data analysis and writing the manuscript. MT K supervised conjugation and molecular experiments. JEC provided J53 *E. coli *and experimental procedures. GEF provided phenotypically identified bacterial isolates. GMM (senior author) designed experimental settings, finalized data analysis and the writing of the manuscript. All authors read and approved the final manuscript.
